# Complete countrywide mortality in COVID patients receiving ECMO in Germany throughout the first three waves of the pandemic

**DOI:** 10.1186/s13054-021-03831-y

**Published:** 2021-11-29

**Authors:** Christian Karagiannidis, Arthur S. Slutsky, Thomas Bein, Wolfram Windisch, Steffen Weber-Carstens, Daniel Brodie

**Affiliations:** 1 Department of Pneumology and Critical Care Medicine, ARDS and ECMO Centre, Cologne-Merheim Hospital, Kliniken Der Stadt Köln gGmbH, Witten/Herdecke University Hospital, Ostmerheimer Strasse 200, 51109 Cologne, Germany; 2 Keenan Research Centre for Biomedical Science, Li Ka Shing Knowledge Institute, St Michael’s Hospital; University of Toronto, Toronto, Canada; 3 Faculty of Medicine, University of Regensburg, Regensburg, Germany; 4 Department of Anesthesiology and Operative Intensive Care Medicine (CCM, CVK), Charité - Universitätsmedizin Berlin, Berlin, Germany; 5 Department of Medicine, Columbia University College of Physicians and Surgeons, and the Center for Acute Respiratory Failure, New York-Presbyterian Hospital, New York, USA

## Research Letter

Extracorporeal membrane-oxygenation (ECMO) has been widely used for COVID-19-related acute respiratory distress syndrome (ARDS), with a mortality rate of 37.1% based on the largest published series [1]. This rate is comparable to ECMO-supported patients with non-COVID-19-related ARDS [1]. However, some reports suggest that the real-life mortality is higher than that reported above [2], including a cohort of 768 COVID-19 patients in Germany with an in-hospital mortality rate of 73% [3], and a surprising finding is given that health care resources in Germany were not notably under strain during the pandemic. To better characterize this discrepancy, we evaluated in-hospital mortality for all COVID-19 patients in Germany supported with venovenous ECMO (VV-ECMO).

We report unbiased and unselected follow-up data at hospital discharge from the federal German hospital payment institute (InEK) of all 3.397 COVID-19 patients supported with VV-ECMO in Germany from March 1^st^, 2020, through May 31^st^, 2021. ECMO in Germany was provided in 213 intensive care units (ICUs)—out of a total of 1.684 intensive care units across 1.288 hospitals (www.intensivregister.de). The study was approved by the Ethics Committee of the Witten/Herdecke University.

**Fig. 1:**
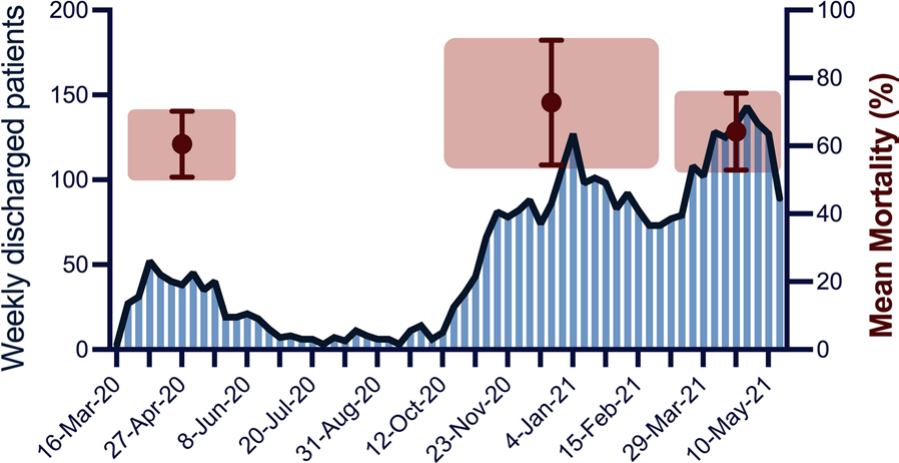
Weekly patient number of patients being treated with VV-ECMO in Germany for COVID-19-related acute respiratory failure between March 2020 and May 2021. Mean mortality is given for all three waves with mean and standard deviation

The mean age of all ECMO patients remained stable over the study period (57 ± 11 years). As expected, survivors were younger than the non-survivors (53 years (range 47–63 y) vs. 59y (Range: 54-63y)), independent of timing during the pandemic. The mean duration of ECMO support was not different between survivors and non-survivors, averaging 17 days. Overall, in-hospital mortality was 68%. Of note, the mortality for any given week (Fig. 1) shows some degree of variation, as survivors spend more time in hospital than non-survivors.

In the largest unbiased and unselected real-life cohort reported to date of VV-ECMO-supported patients with COVID-19-related respiratory failure, we found that in-hospital mortality for patients in Germany was 68%. This is markedly higher than that reported from other countries or from international registries [2–5], confirming prior results [3] of a higher mortality rate in Germany than reported elsewhere.

Mortality in ECMO-supported COVID-19 patients may be increasing over the course of the pandemic, as reported in the most recent Extracorporeal Life Support Organization (ELSO) cohort with a mortality of 51.9% in patients initiated on ECMO after May 1, 2020 [5]. However, that is still lower than the present cohort from Germany. One explanation is the older mean age of 57 ± 11 years in our report compared to the ELSO report (median 51 years). Another explanation is that the use of ECMO in Germany is not centrally regulated and clinicians could elect to initiate ECMO without constraints from regulatory bodies, potentially extending criteria beyond those patients who would be likely to benefit. Furthermore, in Germany, there is an incentive system for the control of health care which is characterized by proportional reimbursement: “as more procedures are done, as more will be paid by insurances,” probably leading to a mixture of ‘true’ indications and reimbursement temptations [6]. A comprehensive central case registry would be particularly important during a pandemic to frequently analyze data and draw updated conclusions. Finally, the ELSO data represent dedicated ECMO centers, whereas the current data are unselected from all German hospitals.

A major strength of the study is inclusion of every COVID-19 patient treated with VV-ECMO in Germany (population ~ 83,000,000) over the course of the study. One limitation is the lack of granularity of the data, including comorbidities, impairing our ability to better understand the reasons for the high mortality.

Ultimately, the present data should serve as a warning to clinicians. Even in a country with adequate resources, mortality in ECMO-supported patients with COVID-19-related respiratory failure may be high if its use is not restricted to patients deemed most likely to benefit.

## Data Availability

Not applicable.
